# Twist1 is highly expressed in cancer-associated fibroblasts of esophageal squamous cell carcinoma with a prognostic significance

**DOI:** 10.18632/oncotarget.17941

**Published:** 2017-05-17

**Authors:** So-Young Yeo, Sang-Yun Ha, Keun-Woo Lee, Yan Cui, Zhao-Ting Yang, Yan-Hua Xuan, Seok-Hyung Kim

**Affiliations:** ^1^ Department of Pathology and Translational Genomics, Samsung Medical Center, Sungkyunkwan University School of Medicine, Seoul, Republic of Korea; ^2^ Department of Oncology, Affiliated Hospital of Yanbian University, Yanji, China; ^3^ Key Laboratory of Natural Resources of the Changbai Mountain and Functional Molecules, Ministry of Education, Yanbian University, Yanji, China; ^4^ Department of Pathology, Yanbian University College of Medicine, Yanji, China; ^5^ Department of Health Science and Technology, Samsung Advanced Institute for Health Science and Technology, Sungkyunkwan University, Seoul, Republic of Korea

**Keywords:** Twist1, cancer associated fibroblast, stroma, esophageal squamous cell carcinoma

## Abstract

Cancer-associated fibroblasts (CAFs) play important roles in cancer progression. Twist1 was recently reported to be a key regulator of CAFs in gastric cancer, but its role in other types of cancer remains unclear, especially for esophageal squamous cell carcinoma (ESCC). We assessed the Twist1 expression on stromal fibroblasts using immunohistochemistry in 169 tissue specimens from ESCC patients, and performed *in vitro* and *in vivo* experiments to confirm the role of Twist1 in CAFs of ESCC. And we investigated the biological pathways that are activated in Twist1-high ESCC using The Cancer Genome Atlas (TCGA) data. The expression of Twist1 in stromal fibroblasts was observed in 89.9% of ESCC patients and positively associated with the increased depth of tumor invasion, lymph node metastasis, and advanced clinical stage, and a significant adverse prognostic factor in overall survival. Twist1-expressing stromal fibroblasts also expressed representative CAF markers, and co-localization of Twist1 and CAF markers were confirmed by confocal immunofluorescence imaging. Bioinformatic analysis of mRNA expression data of esophageal cancer from TCGA revealed that gene sets of CAFs were highly enriched in Twist1-high ESCC. Depletion of *Twist1* in *ex vivo* cultured ESCC CAFs induced significant decrease in migration, invasion, colony formation, sphere formation, and contractibility of ESCC cancer cells compared to control CAFs. Furthermore, Twist1-expressing fibroblasts remarkably enhanced the *in vivo* tumorigenicity of ESCC in a xenograft model. In conclusion, Twist1 could be a novel CAF marker for the prognostic evaluation of ESCC patients as well as a potent therapeutic target for ESCC.

## INTRODUCTION

Activated fibroblasts that are recruited into cancer tissue, called cancer-associated fibroblasts (CAFs), promote tumor progression via a variety of methods, including secreting cytokines and remodeling of extracellular matrix [1]. CAFs mainly contribute to the invasive process by inducing the epithelial–mesenchymal transition (EMT) of tumor cells, a known epigenetic program leading cancer cells to engage a mesenchymal, motile and proteolytic phenotype [2, 3]. CAFs are often characterized by the expression of platelet-derived growth factor α (PDGFRα), PDGFRβ, smooth muscle actin (SMA), fibroblast activation protein (FAP) and fibroblast-stimulating protein-1 (FSP1) in ESCC. However, these markers are typically expressed only in a fraction of fibroblasts within the tumor, and are not specific to CAFs [4]. This large heterogeneity in marker expression for CAF subpopulations or in CAFs originating from different tumors may be explained by their possible miscellaneous origin.

Transcription factor Twist1 belongs to the basic helix-loop-helix super family. Twist1 is essential for mesoderm specification and differentiation during development [5]. We showed that Twist1 expression on cancer cells induces the EMT and plays a critical role in ESCC metastasis and poor outcome [6]. However, the expression of Twist1 in stromal fibroblasts within ESCC tissue has not yet been examined. Furthermore, associations between Twist1 and other CAF markers and their clinical significance remain almost completely unknown.

In this study, we evaluated the expression of Twist1 and CAF markers in 169 human ESCC samples and 20 adjacent non-tumor samples using immunohistochemical staining on tissue microarray slides. In particular, we focused on the clinicopathological significance of Twist1 expression and evaluated the relationship between Twist1 and CAF marker expression in ESCC stromal tissues. Furthermore, the prognostic value of Twist1 and CAF marker expression for human ESCC was evaluated using Cox regression and Kaplan-Meier analysis. We also assessed the effect of Twist1 on the tumor-promoting ability of esophageal CAFs and normal fibroblasts (NFs) through *in vitro* and *in vivo* experiments.

## RESULTS

### Twist1 was more abundantly expressed in stromal fibroblasts than in cancer cells of ESCC

**Figure 1:**
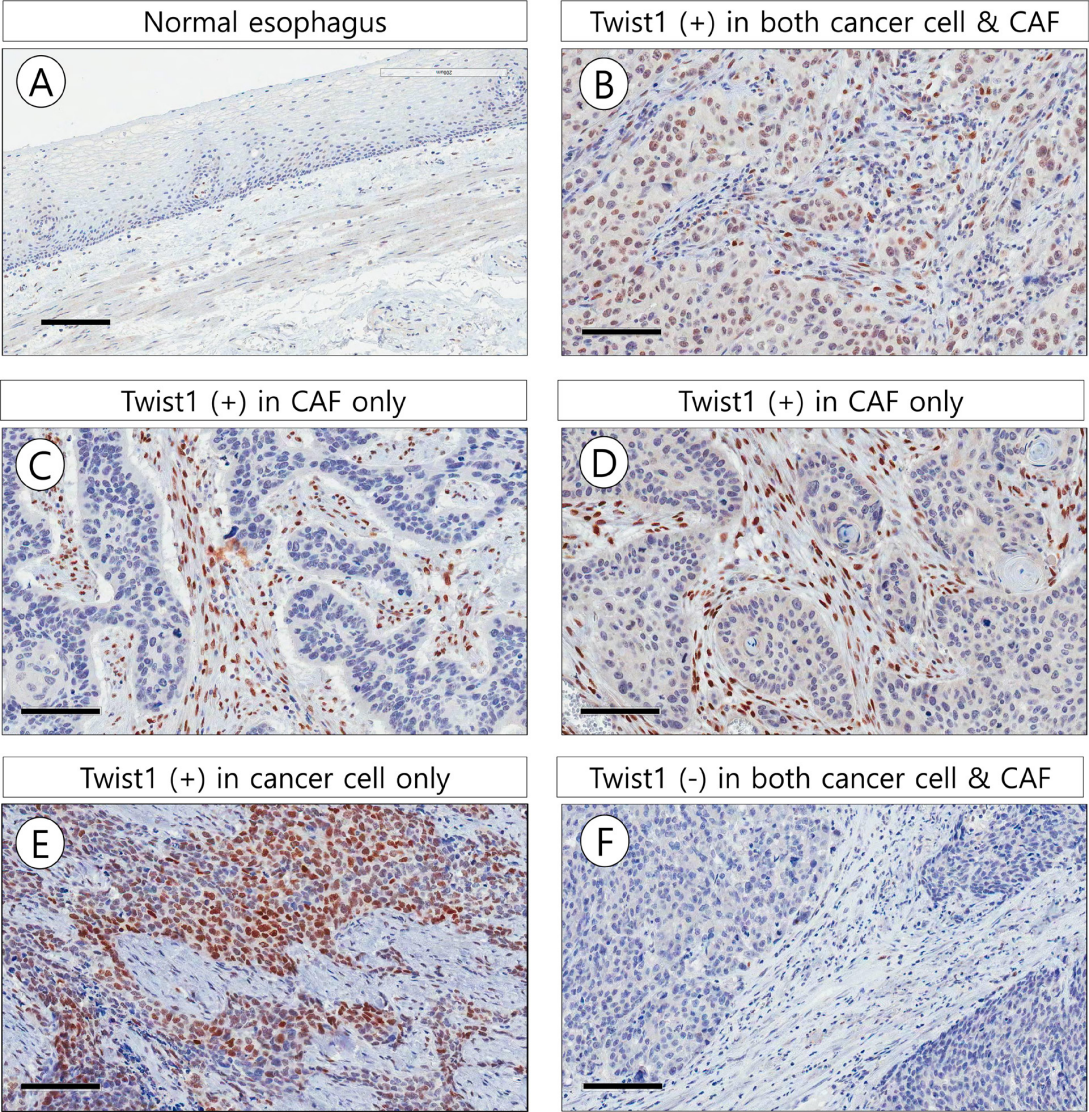
Representative microscopic photographs of Twist1 expression in non-tumor esophageal squamous epithelium (**A**), esophageal squamous cell carcinoma (**B–F**). In 54 cases (Total, 169 cases), Twist1 was expressed in both esophageal cancer cell and cancer associated fibroblast (CAF) (B). In 98 out of 169 cases, Twist1 was expressed in CAF but not cancer cells (C, D). In 2 of 169 cases, Twist1 was expressed in cancer cells but not CAF (E). In 15 out of 169 cases, Twist1 was not expressed in both cancer cells and CAF (F). Scale bar indicates 100 μm.

Twist1 expression rates in stromal fibroblasts were significantly higher in ESCC (152/169, 89.9%) than in adjacent non-tumor esophageal epithelial stroma (8/20, 40.0%) (χ^2^ = 34.338, *P* < 0.001). Positive signals of Twist1 were mainly localized in the nucleus of cancer cells and stromal fibroblasts. Twist1 expression was more frequently identified in ESCC stromal fibroblasts (89.9%) than in cancer cells (33.7%) (χ^2^ = 113.143, *P* < 0.001). In particular, Twist1-expressing stromal cells with a typical mesenchymal phenotype were mostly distributed in the stroma close to cancer cell nests (Figure 1).

Of the 169 cases analyzed, 55 (32.5%) showed positive Twist1 expression in both cancer cells and stromal fibroblasts, 15 (8.9%) showed negative Twist1 expression in both cancer cells and stromal fibroblasts, 2 (1.2%) showed positive Twist1 expression in cancer cells only, and 97 (57.4%) showed positive Twist1 expression in stromal fibroblasts only. Interestingly, Twist1 expression in stromal fibroblasts was positively correlated with Twist1 expression in cancer cells (χ^2^ = 4.015, *r* = 0.154, *P* = 0.045).

### Twist1 expression in stromal fibroblasts was strongly associated with unfavorable clinicopathological parameters and reduced survival

Twist1 expression in stromal fibroblasts was associated with advanced pT stage (*P* = 0.007), lymph node metastasis (*P* = 0.030), and advanced clinical stage (*P* = 0.019) (Table 1).

**Table 1:** Comparison of clinicopathologic characteristics according to Twist1 expression status in stromal fibroblasts of esophageal squamous cell carcinoma

Variable	Number	Twist1 (+) *n*(%)	χ^2^	*P*
Age				
< 65 y	38	34 (89.5)	0.012	0.913
≥ 65 y	131	118 (94.1)		
Sex				
male	163	146 (89.6)	0.696	0.404
female	6	6 (100.0)		
Size				
< 5 cm	108	94 (87.0)	2.789	0.095
≥ 5 cm	61	58 (95.1)		
Differentiation				
well	27	26 (96.3)	0.194	0.907
moderate	111	99 (89.2)		
poorly	31	27 (87.1)		
T stage				
Early T stage (T1)	36	28 (77.8)	7.380	0.007
Advanced T stage (T2–4)	133	124 (93.2)		
Lymph node metastasis				
negative	68	57 (83.8)	4.706	0.030
positive	101	95 (94.1)		
Distant metastasis				
negative	147	131 (89.1)	0.757	0.384
positive	22	21 (95.4)		
Clinical stage				
Early (stage1)	26	20(76.9)	5.526	0.019
Advanced (stage 2–4)	143	132(92.3)		
Chemotherapy				
negative	57	48(84.2)	2.156	0.142
positive	112	104 (92.9)		
Radiotherapy				
negative	99	88 (91.7)	0.532	0.466
positive	70	64 (87.7)		
Epithelial to mesenchymal transition (EMT)				
Complete	37	36 (97.3)	2.923	0.232
Incomplete	45	39 (86.7)		
No EMT	87	78 (89.7)		

**Figure 2:**
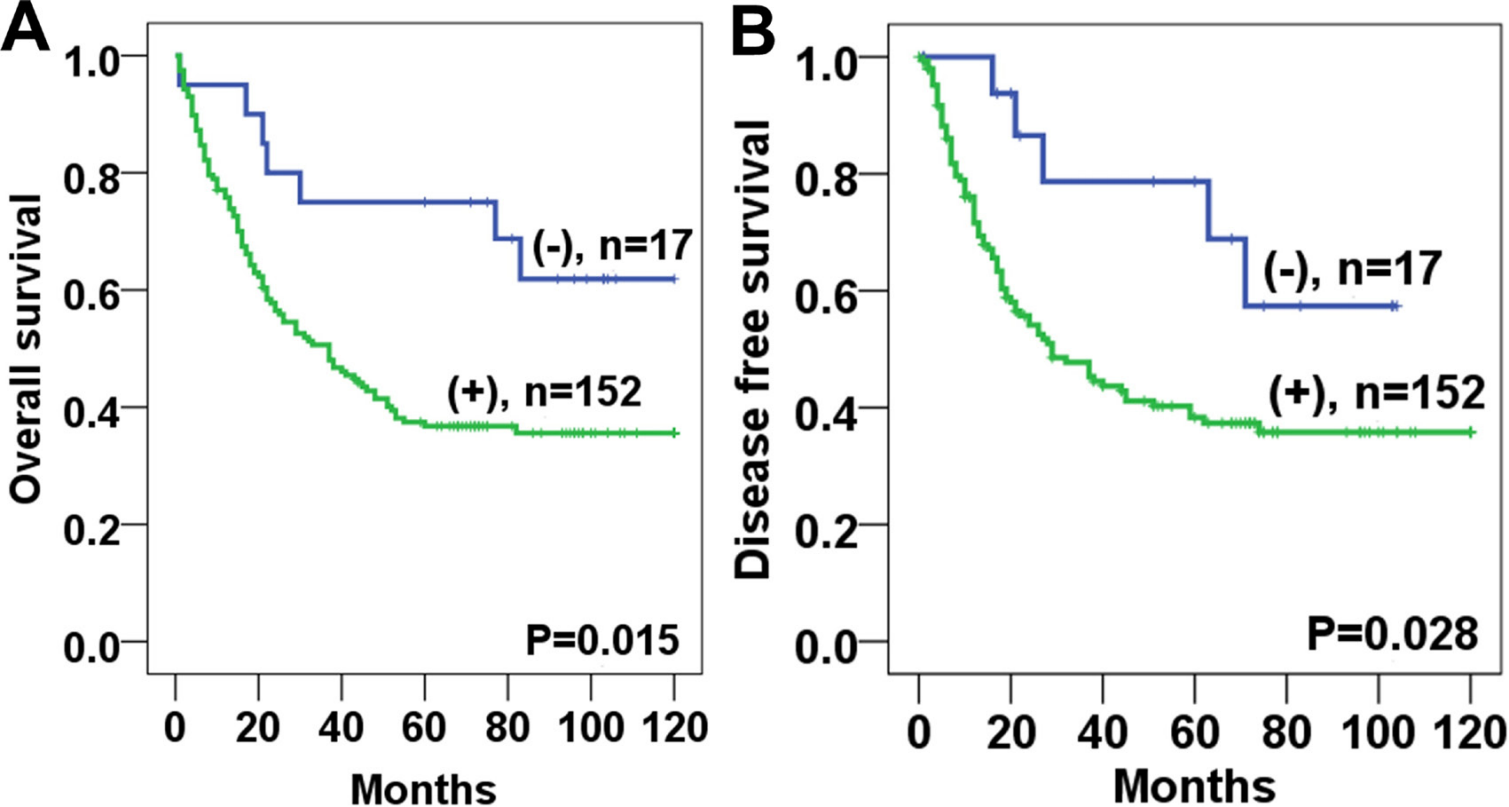
Kaplan-Meier analyses of overall survival and disease free survival curves for Twist1 expression in esophageal squamous cell carcinoma stromal tissues patients Patients with Twist1 positive expression in stromal fibroblast had lower overall survival (**A**) and disease free survival (**B**) rates than those with Twist1 negative expression.

Survival analysis revealed that Twist1 expression in stromal fibroblasts was significantly associated with poor overall survival (OS) (*P* = 0.015) and disease-free survival (DFS) (*P* = 0.028) (Figure 2). The 5-year OS and DFS rates of the Twist1-positive group (36.9%, 44.0%, respectively) were significantly lower than those of the Twist1-negative group (65.0%, 72.2%, respectively). In the univariate Cox regression analysis, Twist 1 expression was a poor prognostic factor for both OS and DFS (Table 2). In addition, Twist1 expression in stromal fibroblasts was an independent predictor of poor prognosis for OS (HR: 2.78, *P* = 0.010) in the multivariate analysis (Table 3).

**Table 2:** Univariate analyses for prognostic variables of overall survival and disease-free survival in ESCC patients using Cox proportional-hazards regression

Characteristic	Overall survival			Disease–free survival		
	HR	95% CI	*p*–value	HR	95% CI	*p*–value
**Age (years)**			0.213			0.769
< **65**	1.00		–	1.00		–
≥ **65**	1.362	0.837–2.217		0.929	0.569–1.518	
**Tumor size (cm)**			0.228			0.101
< **4**	1.00		–	1.00		–
≥ **4**	1.274	0.860–1.887		1.424	0.934–2.172	
**T stage**			0.001			0.002
**1**	1.00		–	1.00		–
**2–4**	2.536	1.443–4.454		3.011	1.510–6.003	
**Lymph node metastasis**			0.005			< 0.001
**Negative**	1.00		–	1.00		–
**Positive**	1.767	1.184–2.637		2.655	1.637–4.308	
**Distant metastasis**			0.049			< 0.001
**Negative**	1.00		–	1.00		–
**Positive**	1.735	1.003–3.001		2.637	1.566–4.441	
**Twist1 in CAF**			0.003			0.019
**Negative**	1.00		–	1.00		–
**Positive**	3.252	1.508–7.016		2.523	1.164–5.467	

**Table 3:** Multivariate analyses for prognostic variables of overall survival and disease-free survival in ESCC patients using Cox proportional-hazards regression

Characteristic	Overall survival			Disease–free survival		
	HR	95% CI	*p*–value	HR	95% CI	*p*–value
**Age (years)**			0.172			0.548
< **65**	1.00		–	1.00		–
≥ **65**	1.407	0.862–2.299		0.858	0.521–1.414	
**Tumor size (cm)**			0.978			0.551
< **4**	1.00		–	1.00		–
≥ **4**	0.994	0.664–1.489		1.141	0.739–1.762	
**T stage**			0.027			0.067
**1**	1.00		–	1.00		–
**2–4**	1.974	1.081–3.603		1.997	0.954–4.183	
**Lymph node metastasis**			0.255			0.019
**Negative**	1.00		–	1.00		–
**Positive**	1.286	0.834–1.984		1.871	1.110–3.154	
**Distant metastasis**			0.245			0.009
**Negative**	1.00		–	1.00		–
**Positive**	1.399	0.795–2.461		2.036	1.193–3.474	
**Twist1 in CAF**			0.010			0.108
**Negative**	1.00		–	1.00		–
**Positive**	2.780	1.277–6.051		1.910	0.869–4.200	

### Twist1-expressing stromal fibroblasts also expressed representative CAF markers

**Figure 3:**
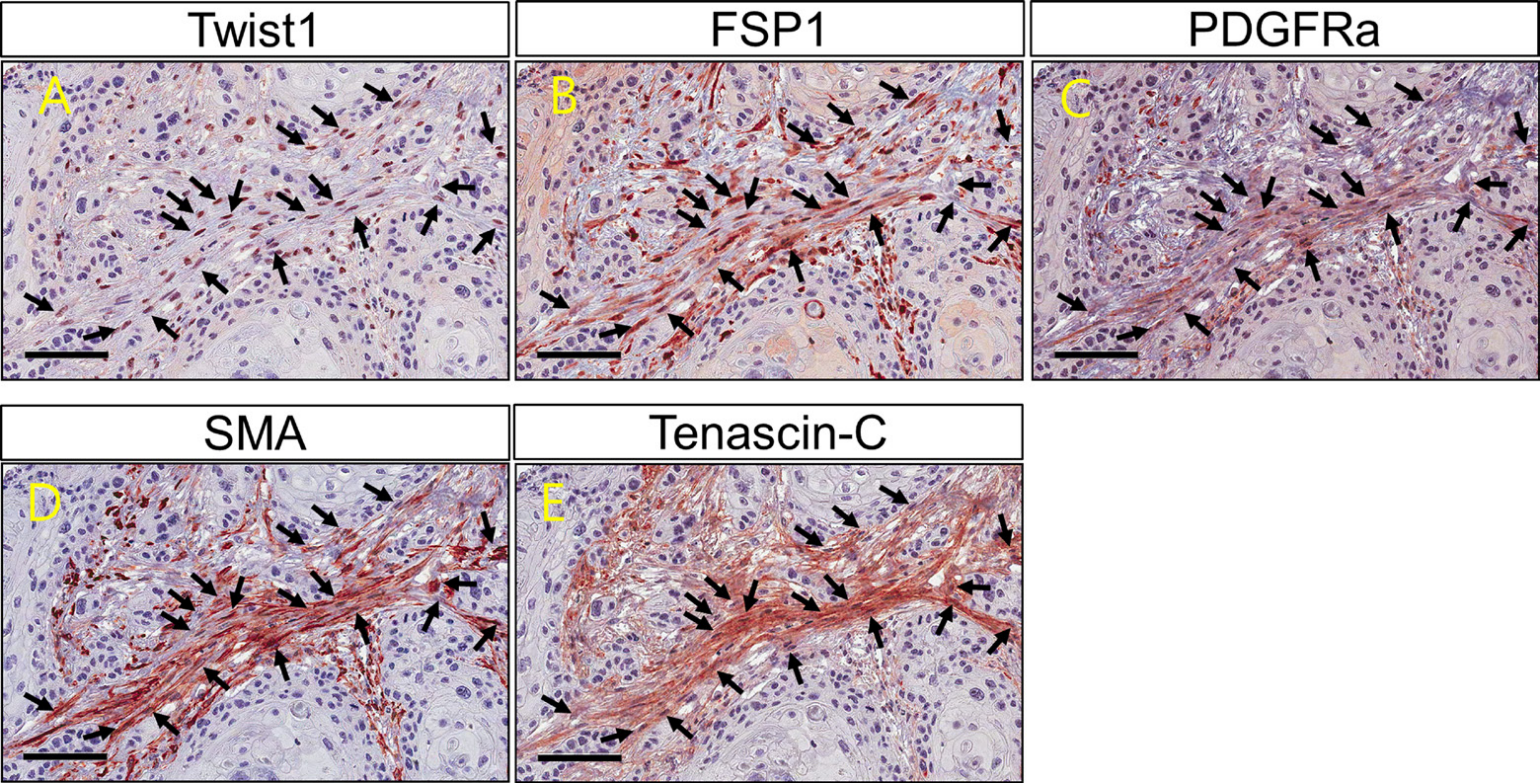
Twist1 and cancer associated fibroblast markers (FSP1, PDGFRα, SMA and Tenascin C) expression on fibroblasts within cancer stroma Co-expression of Twist1 and cancer associated fibroblast markers (FSP1, PDGFRα, SMA and Tenascin C) is observed in the individual stromal fibroblasts (arrow).

**Figure 4:**
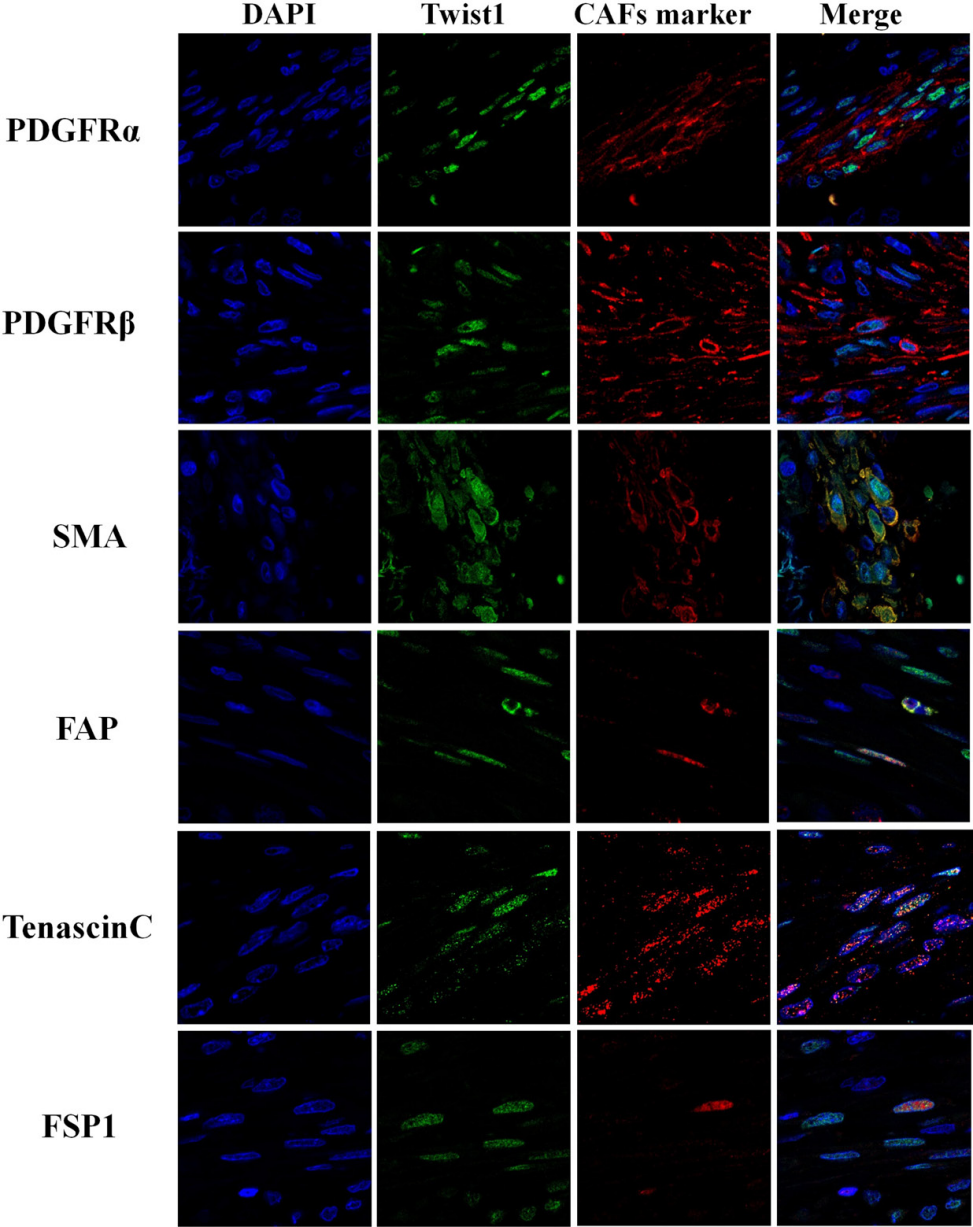
Immunofluorescence analysis of Twist1 and cancer associated fibroblast markers (PDGFRα, PDGFRβ, SMA, FAP, Tenascin C and FSP1) in stromal fibroblast of esophageal squamous cell carcinoma

Twist1 expression in ESCC stromal fibroblasts was positively correlated with PDGFRα (*P* = 0.009), SMA (*P* = 0.003) and Tenascin-C (*P* = 0.001) expression (Table 4). Moreover, the co-expression of Twist1 and CAF markers was examined in individual cancer-stromal fibroblasts of five ESCC tissue samples using confocal microscopy. Twist1-expressing stromal fibroblasts co-expressed representative CAF markers such as PDGFRα, SMA, and Tenascin-C (Figures 3 and 4), morphologically and phenotypically identified them as CAFs. Furthermore, lymph node metastasis of ESCC occurred more frequently when stromal fibroblasts were positive for both Twist1 and CAF markers. Specifically, fibroblast positivity for both Twist1 and Tenascin-C (*P* = 0.003), Twist1 and SMA (*P* = 0.007), and Twist1 and FSP1 (*P* = 0.004) were each strongly correlated with enhanced nodal metastasis compared with controls in which stromal fibroblasts were negative for either Twist1 or CAF markers (Table 5) .

**Table 4:** The association between protein expression of Twist1 and that of cancer associated fibroblast markers in ESCC stromal fibroblasts

**Variable**	***n***	**Twist1 (+) *n* (%)**	**χ**^2^	**R**	***P***
FSP1					
negative	43	37 (86.0)	0.735	0.074	0.395
positive	126	115 (91.3)			
SMA					
negative	34	26 (76.5)	8.737	0.233	0.003
positive	135	126 (93.3)			
Tenascin C					
negative	52	41 (78.8)	10.017	0.273	0.001
positive	117	111 (94.9)			
PDGFRα					
negative	20	15 (75.0)	6.798	0.225	0.009
positive	149	137 (91.9)			

**Table 5:** The Lymph node metastasis rates of patients with expression of Twist1 and cancer associated fibroblast markers in stromal fibroblasts of esophageal squamous cell carcinoma

Lymph node metastasis	Twist1&FSP1			**χ**^2^	*P*	Twist1&PDGFα			**χ**^2^	*P*	Twist1&SMA			**χ**^2^	*P*	Twist1&Tenascin C			**χ**^2^	*P*
	*n*	both (−)	both (+)			*n*	both (−)	both (+)			*n*	both (−)	both (+)			*n*	both (−)	both (+)		
		*n* (%)	*n* (%)				*n* (%)	*n* (%)				*n* (%)	*n* (%)				*n* (%)	*n* (%)		
negative	35	6 (17.1)	29 (82.9)	8.209	0.004	47	5 (10.6)	42 (89.4)	1.899	0.168	41	6 (14.6)	35(85.4)	7.347	0.007	33	7 (21.2)	26 (78.8)	8.800	0.003
positive	63	1 (1.6)	62 (98.4)			72	3 (4.2)	69 (95.8)			88	2 (2.3)	86(97.7)			66	2 (3.0)	64 (97.0)		

Both (−): negative for both markers, Both(+): positive for both markers.

### Double positivity for both Twist1 and CAF markers in stromal fibroblasts was strongly correlated with reduced OS and DFS

**Figure 5:**
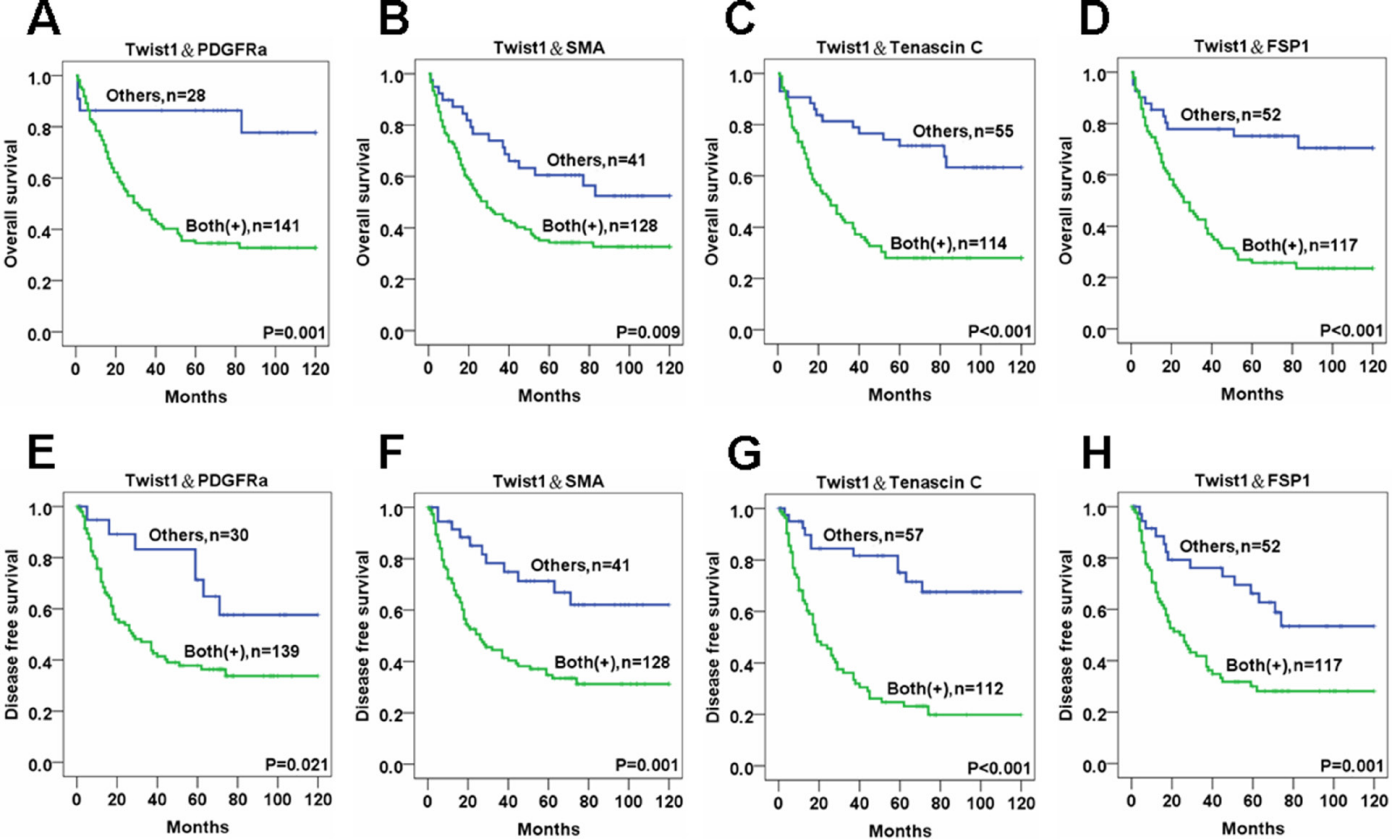
The survival analysis of esophageal cancer patients for the expressions of Twist1 and various CAF markers in cancer stromal fibroblasts Overall and disease free survival rates revealed by Kaplan-Meier survival analysis are lower in ESCC patients whose stromal fibroblasts are positive for both Twist1 and PDGFRα (**A, E**), Twist1 and SMA (**B, F**), Twist1 and Tenascin C (**C, G**), and Twist1 and FSP1 (**D, H**) than other patients.

OS and DFS rates were markedly lower for ESCC patients with stromal fibroblasts that expressed both Twist1 and one CAF marker compared to controls. Specifically, double positivity in ESCC stromal fibroblasts for Twist1 and PDGFRα (OS:*P* = 0.001 and DFS:*P* = 0.021), Twist1 and SMA (OS:*P* = 0.009 and DFS:*P* = 0.001), Twist1 and Tenascin-C (OS:*P* < 0.001 and DFS:*P* < 0.001) and Twist1 and FSP1 (OS:*P* < 0.001 and DFS:*P* = 0.001) were all very strongly correlated with reduced OS and DFS compared with controls with stromal fibroblasts that were negative for Twist1 and CAF markers (Figure 5). In addition, patients with ESCC showing expression of all 5 CAF markers (Twsit1, FSP1, SMA, Tenascin C, PDGFRa) showed significantly reduced OS and DFS rate than other patients (Supplementary Figure 1).

### Analysis of TCGA data revealed correlations between Twist1 and CAF markers as well as enrichment of CAF-related gene signatures in Twist1-high ESCC

**Figure 6:**
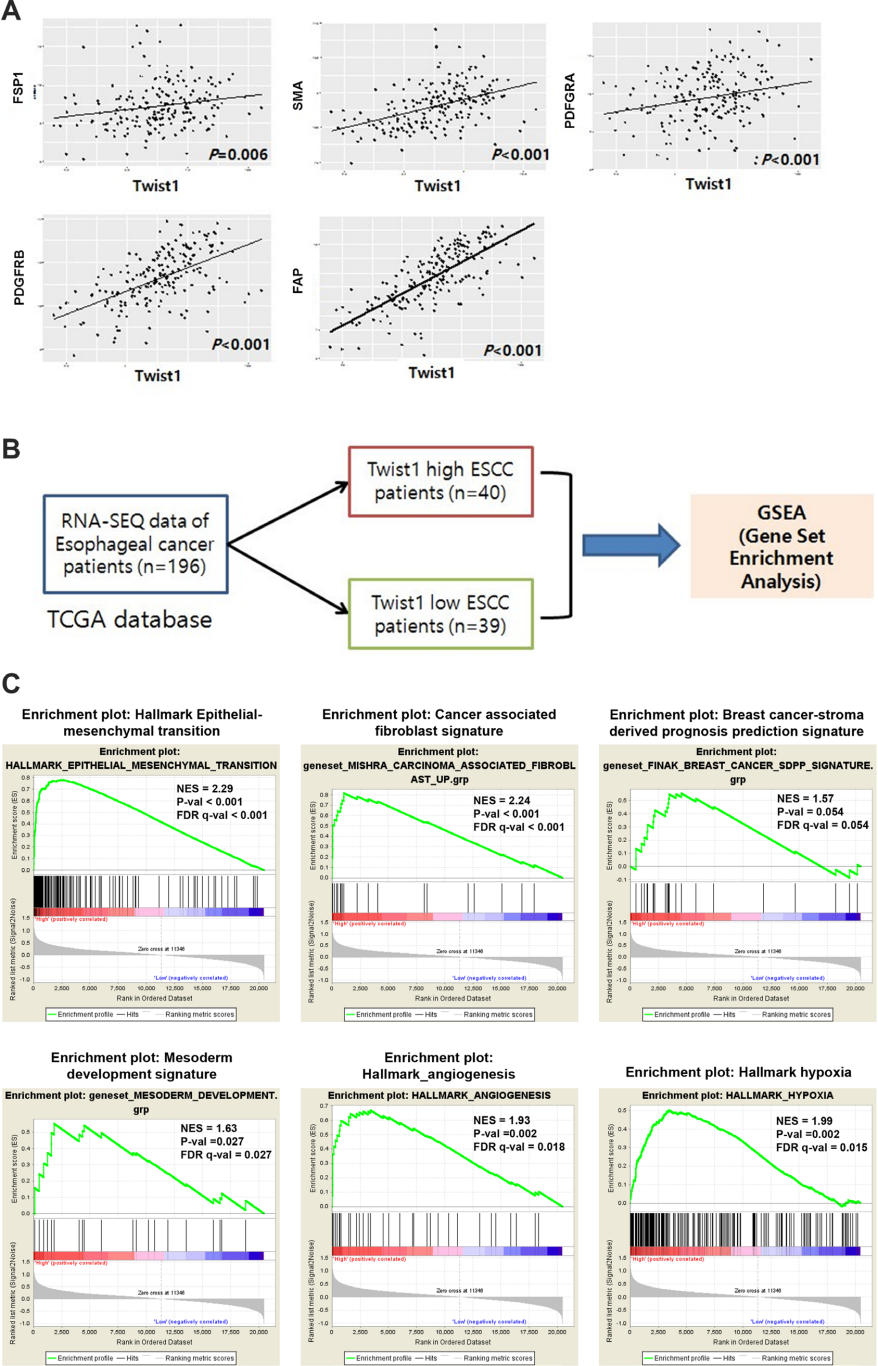
Analysis of TCGA data revealed correlation between Twist1 and CAF markers as well as enrichment of CAF related gene signatures in Twist1-high ESCC (**A**) Correlation of mRNA level of Twist1 with CAF markers such as FSP1, PDGFRα, PDGFRβ, FAP, SMA in esophageal cancer. These results are obtained from TCGA ESCA data. (**B**) Schematic illustration of gene set enrichment analysis (**C**) The gene sets of key mesenchymal signatures are enriched in Twist1 high esophageal cancers were enriched.

To validate these results with an independent data source and further investigate biological pathways active in Twist1-high ESCC, we analyzed normalized gene expression data for 196 esophageal cancers (RNA sequencing) downloaded from TCGA. We found that the mRNA level of Twist1 was strongly correlated with levels of CAF markers, especially PDGFRB, SMA, and FAP (Figure 6). These results were highly consistent with data obtained through immunohistochemical studies (Figure 6) and demonstrated that this TCGA ESCC dataset was suitable for further study. Next, we performed gene set enrichment analysis (GSEA) on a Twist1-high esophageal cancer group from the cohort of 196 esophageal cancers with TCGA data. GSEA was performed using the Broad Institute’s GSEA tool. The expression of Twist1 was strongly associated with the gene set of the Epithelial-Mesenchymal Transition (NES = 2.29, FDR *q*-val < 0.001), as expected (Figure 6). In addition, gene signatures related to activated cancer stroma were also strongly enriched in Twist1-high ESCC patients, including gene sets of carcinoma-associated fibroblasts (NES = 2.24, FDR *q*-val < 0.001), angiogenesis (NES = 1.93, FDR *q*-val = 0.018), and hypoxia (NES = 1.99, FDR *q*-val = 0.015) (Figure 6). Moreover, we found that the gene signature of breast cancer-stroma-derived prognosis prediction was also marginally upregulated in Twist1-high ESCC (NES = 1.57, FDR *q*-val = 0.054). Most curiously, gene sets of mesoderm development were also significantly enriched (NES = 1.63, FDR *q*-val = 0.027) in Twist1-high esophageal cancer. Altogether, these results suggest that Twist1 is strongly associated with not only the EMT, but also cancer-associated fibroblasts, in ESCC.

### Twist1 is a necessary and sufficient condition for the expressions of CAF markers in *ex vivo* cultured esophageal CAFs and normal fibroblasts

**Figure 7:**
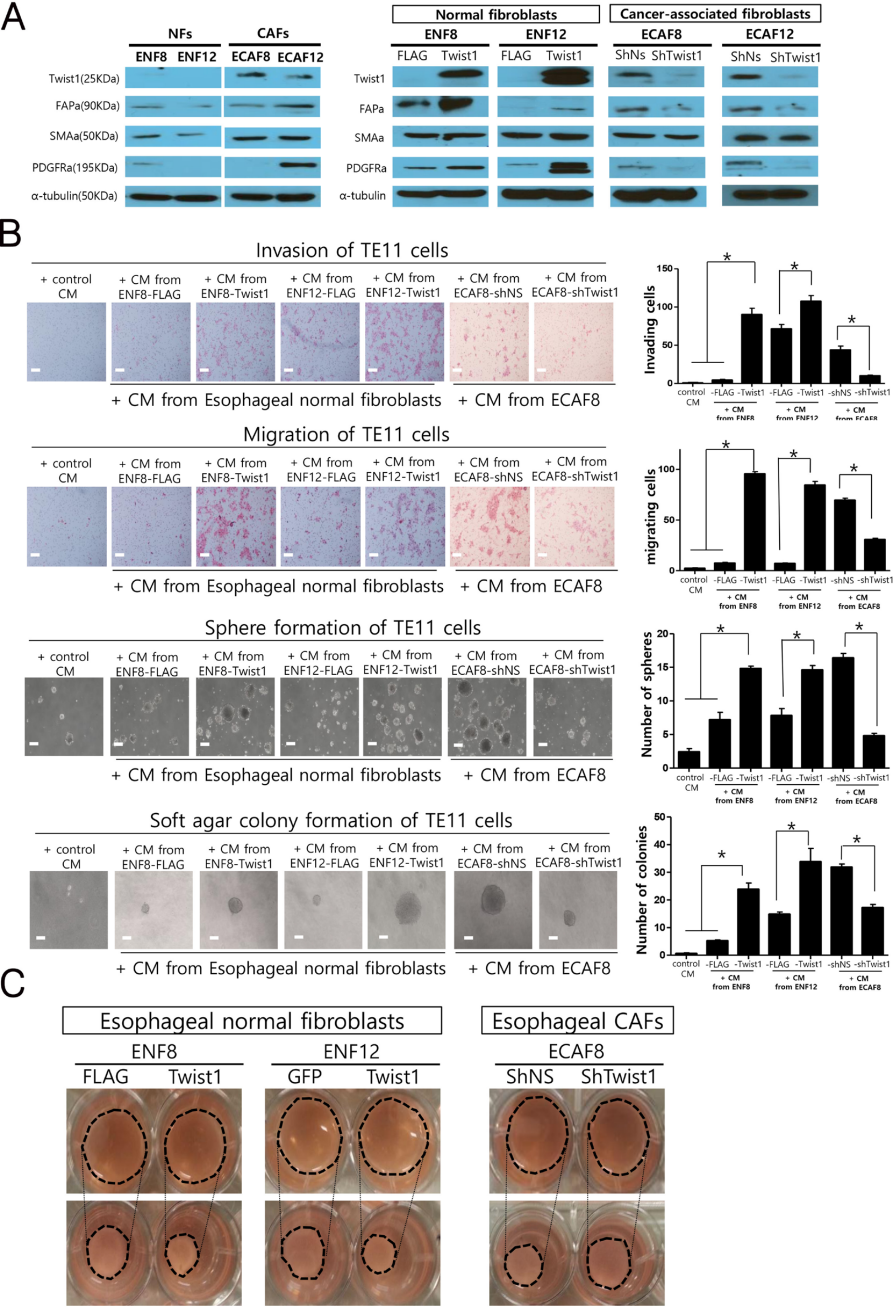
The *in vitro* effects of Twist1 expression in esophageal cancer-associated fibroblasts (CAFs) and normal fibroblasts (NFs) on the progression of esophageal cancer cells and the contraction ability of fibroblasts (**A**) Twist1 and CAF markers expression in NFs and CAFs by western blotting analysis. (**B**) The migration, invasion, sphere formation and soft agar colony formation of TE11 cell lines (esophageal cancer cells) were significantly increased by treating conditioned media from Twist1 expressing esophageal normal fibroblasts. On the other hand, these *in vitro* parameters showing tumor progressions were decreased by treating conditioned media from Twist1-depleted CAFs. (**C**) Down-regulation and overexpression of Twist1 significantly reduced and increase collagen contraction capacities in esophageal fibroblasts, respectively.

Next, we studied whether Twist1 induces the expressions of various CAF markers in esophageal normal fibroblasts and CAFs. For this study, we established *ex vivo* cultures of two pairs of normal fibroblasts and CAFs from two ESCC tissues. Twist1 expression was observed in CAFs (ECAF8, ECAF12), but not in patient-matched normal fibroblasts (NFs) (ENF8, ENF12), which is highly consistent with our histologic observation shown in Figure 1 (Figure 7A). CAF markers were more strongly expressed in CAFs than in normal fibroblasts (Figure 7A). Then, we increased Twist1 expression in normal fibroblasts (ENF8, ENF12) by transducing with lentiviral particles expressing Twist1. As a result, Twist1 increased the expressions of CAF markers including FAPa, PDGFRa. In contrast, the shRNA-mediated depletion of Twist1 in CAFs (ECAF8, ECAF12) reduced CAF marker expressions (Figure 7A). These results in CAFs are very well consistent with frequent overexpression of Twist1 in CAFs of ESCC.

### Increased expression of Twist1 in esophageal normal fibroblasts enhanced migration, invasion, and sphere formation of esophageal cancer cells, thereby enhancing tumor promoting ability of esophageal fibroblasts

These data mentioned above, strongly suggest that Twist1 is able to enhance the tumor promoting ability of esophageal fibroblast. To prove this hypothesis, we induced the Twist1 expression in esophageal normal fibroblast using lentiviral vector as aforementioned, then we treated TE11 esophageal cancer cells with conditioned media (CM) of fibroblasts. Indeed, the migration and invasion of TE11 cancer cells were significantly increased by CM from Twist1-expressing fibroblasts compared to control fibroblasts. *In vitro* anchorage independent tumorigenic activity of TE11 ESCC cells was also significantly increased in cancer cells treated with CM from Twist1-expressing esophageal fibroblast (ENF8) as shown in sphere formation assay and soft agar colony formation assay (Figure 7B).

### Knockdown Twist1 expression in esophageal CAFs impairs the ability of CAFs to promote tumor, such as facilitating invasion, migration and sphere formation of esophageal cancer cells

On the other hand, shRNA-mediated depletion of Twist1 in esophageal CAFs reduced tumor-promoting ability of CAFs. The migration, invasion, and *In vitro* anchorage independent tumorigenic activity of TE11 esophageal cancer cells were significantly decreased when treated with conditioned media (CM) from shTwist1-treated esophageal CAFs (ECAF8) compared to control group (Figure 7B, 7C). These results strongly suggest that Twist1 enhances tumor promoting ability of esophageal CAF by secreting soluble factors because these effects of CAF on cancer cells behavior were shown using conditioned media containing soluble factors. And these results are well consistent with our clinical observation previously mentioned that Twist1 overexpression was positively correlated with advanced T stage and lymph node metastasis in ESCC, because both indicates the extent and depth of direct tumor invasion (Table 2).

### Twist1 augment esophageal fibroblast-mediated collagen gel contraction

The primary function of fibroblast is to secrete and remodel the extracellular matrix (ECM). In addition to secreting soluble factors, Twist1 may enhance the tumor-promoting ability of CAF by augmenting ECM-remodeling because ECM greatly influences on the behavior of cancer cells. This hypothesis was verified by examining the effect of Twist1 on fibroblast-mediated collagen gel contraction. As expected, Induced Twist1-expression in esophageal normal fibroblast (ENF8) increased fibroblast-mediated collagen gel contraction (Figure 7C). Furthermore, shRNA-mediated down-regulation of Twist1 in esophageal CAF (ECAF8) reduced collagen gel contraction (Figure 7C).

### Twist1 expression in esophageal normal fibroblasts enhanced the mRNA level of Twist1 in esophageal cancer cell

**Figure 8:**
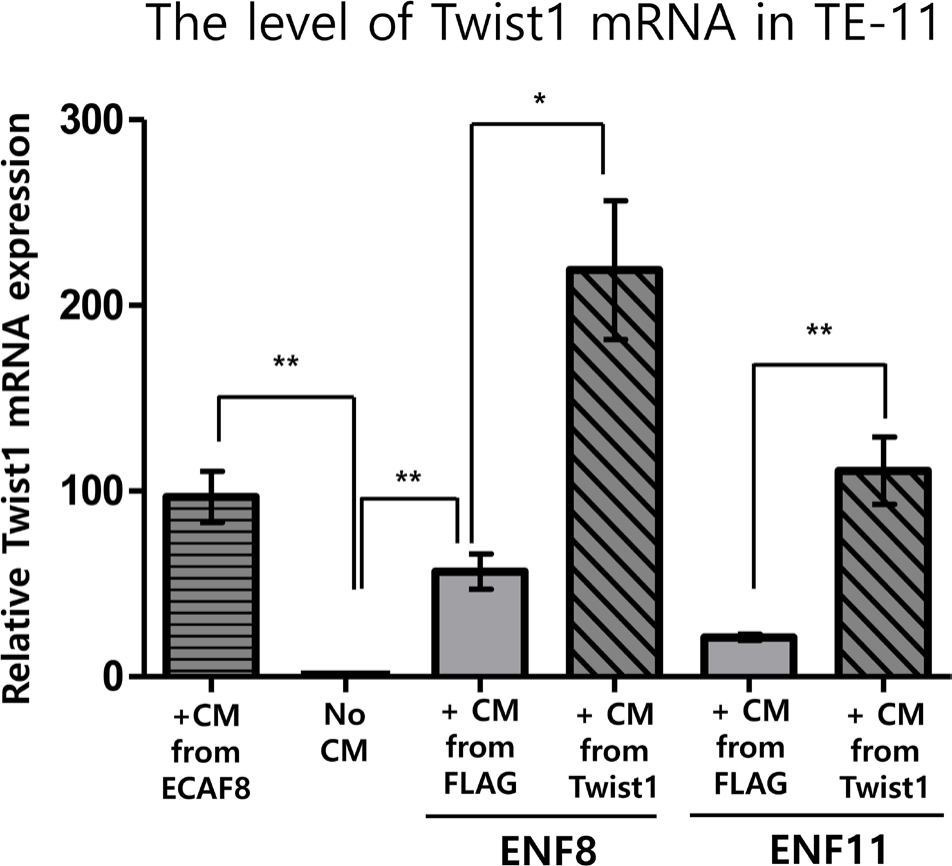
The effect of Twist1 expression in esophageal normal fibroblasts (NFs) on the expression of Twist1 in esophageal cancer cell (TE11) Conditioned medium (CM) obtained from ECAF8 significantly increased Twist1 expression in TE11 cells compared to control (No CM). On the other hand, Induced Twist1 expression (+CM from Twist1) in two esophageal normal fibroblasts (ENF8 and ENF11) also remarkably enhanced mRNA level of Twist1 in TE11 cells compared to controls (+CM from FLAG). For this study, conditioned medium was obtained from normal fibroblasts infected by Twist1-expressing lentivirus (+CM from Twist1). *P < 0.05, **P < 0.01.

As shown above, Twist1 expression in esophageal fibroblast facilitates the migration and invasion of cancer cells. Because Twist1 was reported to induce the migration and invasion of esophageal cancer cells [6], we speculated that Twist1 expressing fibroblasts also increase Twist1 expression in esophageal cancer cells. Indeed, conditioned media obtained from two Twist1-expressing esophageal normal fibroblasts (ENF8 and ENF11) also remarkably enhanced mRNA level of Twist1 in TE11 cells compared to controls (Figure 8). Likewise, conditioned medium obtained from ECAF8 significantly increased Twist1 expression in TE11 cells compared to control (No conditioned media) (Figure 8).

### Up-regulation of Twist1 in esophageal normal fibroblast enhanced *in vivo* tumorigenicity of esophageal cancer cells

**Figure 9:**
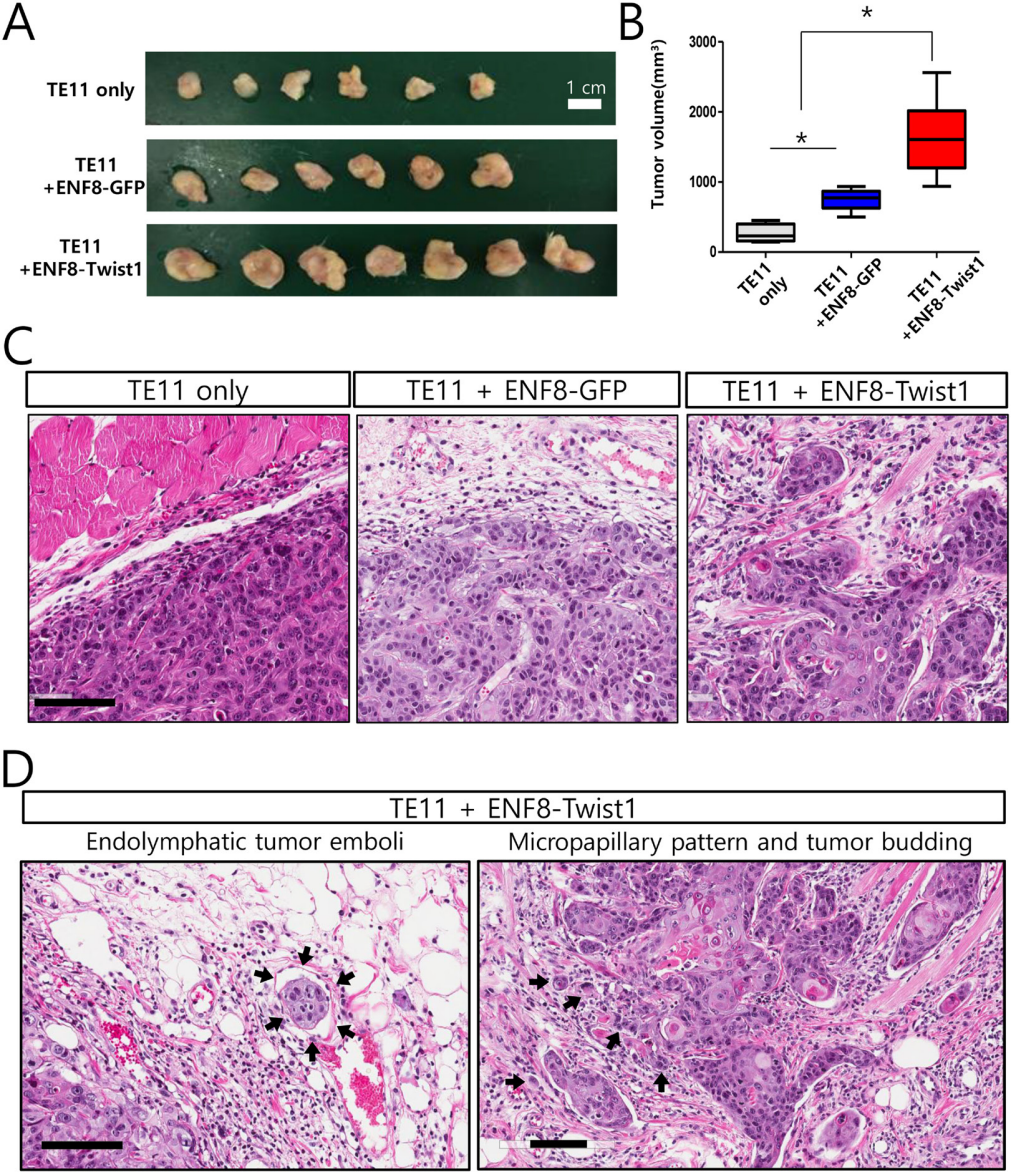
The overexpression of Twist1 in esophageal normal fibroblasts enhanced *in vivo* tumorigenicity of esophageal cancer cells (**A**) TE11 cells were subcutaneously grafted to immune-compromised mice with tumor cells alone, or GFP-expressing esophageal normal fibroblast (ENF8), or Twist1-expressing ENF8. (**B**) Overexpression of Twist1 in esophageal normal fibroblast (ENF8) significantly enhanced the growth of TE11 (ESCC cells) *in vivo*. (**C**) Histologic examination revealed that TE11 xenograft tumor exhibited more infiltrative tumor growth when co-injected with Twist1-expressing esophageal fibroblast rather than ENF8-GFP or tumor cell alone (TE11 only). Scale bar indicates 100 μm. (**D**) Endolymphatic tumor emboli (left panel) and micropapillary pattern with tumor budding (right panel) were observed only in xenograft infused with both esophageal cancer cell (TE11) and Twist1-expressing esophageal fibroblasts (ENF8-Twist1) Scale bar indicates 100 μm.

Both clinical and *in vitro* functional studies indicated that Twist1 enhanced the ability of esophageal fibroblast to promote tumor progression. Based on these data, it was expected that Twist1-expressing fibroblasts may enhance *in vivo* tumorigenicity of esophageal cancer cells. To confirm this conclusion, we coinjected ESCC TE11 cells subcutaneously into six immune-compromised (NOD/SCID) mice with esophageal normal fibroblast (ENF8) transduced with lentivirus expressing either Twist1 or GFP (control). Tumor growths in these mice were followed up for eight weeks and mice were sacrificed to measure the tumor growth. Twist1-expressing esophageal fibroblasts (ENF8-Twist1) significantly enhanced the growth of TE11 ESCC cells compared to control group (*P* = 0.002) (Figure 9A, 9B). Histologic examination revealed that TE11 xenograft tumor exhibited more infiltrative tumor growth into surrounding tissue when they were co-injected with Twist1-expressing esophageal fibroblast (ENF8-Twist1) rather than control fibroblasts (ENF8-GFP) or tumor cell alone (TE11 only) (Figure 9C). In addition, several highly aggressive cancer-specific histologic parameters such as, endolymphatic tumor emboli, micropapillary pattern, and tumor budding were observed only in TE11+ENF8-Twist1 xenografts not in TE11+ENF8-GFP or TE11 only xenografts (Figure 9D).

## DISCUSSION

In this study, we describe Twist1 expression in stromal fibroblasts as a reliable predictor of poor prognosis in ESCC patients. We found a positive correlation between expression of Twist1 and CAF markers such as PDGFRα, SMA, Tenascin-C and FSP1 in ESCC. In addition, the simultaneous expression of Twist1 and other CAF markers was strongly associated with adverse clinical outcome. Besides, we demonstrated that up-regulation of Twist1 in esophageal fibroblasts significant increased the ability of fibroblasts to promote tumor progression, whereas down-regulation of Twist1 in esophageal CAFs impaired their tumor-promoting ability. Thus, our results indicate that Twist1 plays an important role in CAFs of ESCC.

To date, several reports have indicated that Twist1 is a key transcription factor that induces cancer metastasis via initiation of the EMT in a variety of cancers, including ESCC [6–8]. However, we focused on the role of Twist1 in cancer-associated fibroblasts rather than cancer cells. In our previous report, we demonstrated that Twist1 is frequently expressed in cancer-associated fibroblasts in gastric cancer and is associated with poor prognosis [9]. Moreover, we revealed that Twist1 is a key regulator in the transdifferentiation from normal quiescent fibroblasts to CAFs using cancer tissue derived *ex vivo* CAF cultures [10]. Specifically, proinflammatory cytokine IL-6 induces Twist1 expression in normal fibroblasts via STAT3 phosphorylation, and Twist1 then promotes the transdifferentiation of normal fibroblasts to CAFs via activation of a strong tumor-promoting chemokine, CXCL12, as well as suppression of senescence [10].

Recently, Twist1’s role in CAFs has been highlighted [11, 12]. According to García-Palmero et al., Twist1 drives the activation of stromal fibroblasts, leading to tumor progression via remodeling of the extracellular matrix. During this process, palladin and collagen α1(VI) are down-stream targets of Twist1 [12]. Spaeth et al. reported that Twist1 is a key transcription factor for the transdifferentiation of mesenchymal stem cell to CAFs via CD44 [11]. In the present study, we found that Twist1 expression in ESCC stromal fibroblasts was significantly related to advanced pT stage, lymph node metastasis, and advanced clinical stage. Twist1 expression in ESCC stromal fibroblasts was also associated with a poor survival rate. Moreover, this observation was further confirmed by additional *in vivo* and *in vitro* experiments using *ex vivo* esophageal fibroblast culture. These findings suggest that Twist1-expressing CAFs may play an important role in progress of ESCC. Our findings in ESCC samples are highly consistent with our previous experimental study [10].

One of the most significant findings of this study is a significant correlation in stromal expression between Twist1 and CAF markers such as PDGFRα, SMA and Tenascin-C. We also demonstrated co-localization of Twist1 and other CAF markers in individual ESCC stromal fibroblasts by using immunofluorescence imaging. Moreover, the expressions of CAF markers were decreased by down-regulation of Twist1 in CAF cells. On the other hand, co-expression of Twist1 and CAF markers was strongly correlated with nodal metastasis and unfavorable clinical outcomes. These results suggest that the expression of Twist1 along with other CAF markers might be a potential biomarker for predicting clinical outcome and stratifying patient groups for proper therapeutic strategies.

Our results were also corroborated by bioinformatic analysis of gene expression profiles (RNA-SEQ) in esophageal cancer downloaded from TCGA, where there was a strong correlation between mRNA levels of Twist1 and CAF markers. Although TCGA data were not generated specifically from stromal fibroblasts, the overall results are highly consistent with those from our immunohistochemical studies. Given that these CAF markers and Twist1 are predominantly expressed in stromal fibroblasts, the data from TCGA offer an additional source for insights into the functional significance of stromal Twist1 in esophageal cancer. GSEA of gene expression profiles of the Twist1-high group indicated that Twist1 in esophageal cancer was very strongly associated with the gene signature of cancer-associated fibroblasts, cancer stroma, and even mesodermal development. In fact, Twist1 has long been known to be a master gene of morphogenesis via regulation of neural crest migration and mesodermal development during embryogenesis [13]. Accordingly, our ESCC tissue-based study can be concluded to be validated using a completely independent dataset.

The current study uncovered a function of Twist1 in tumor–stromal cell communication, demonstrated that Twist1 could be a useful prognostic marker, and indicated that high Twist1 expression correlated with poor prognosis in ESCC, making Twist1 a solid candidate as a potent therapeutic target for ESCC.

## MATERIALS AND METHODS

### Tissue specimens

A total of 189 formalin-fixed and paraffin-embedded tissue samples, including 169 ESCC and 20 adjacent non-tumor esophageal mucosa, were obtained from the Department of Pathology at Samsung Medical Center (Seoul, Korea) in accordance with protocols approved by the institutional review board. No patient underwent preoperative chemotherapy or radiotherapy. Clinical and pathological reports were reviewed for age, sex, tumor size, histological grade, invasion depth (pT), nodal status (pN), and distant metastasis (pM). pTNM classification was applied according to guidelines from the 2010 American Joint Committee on Cancer staging manual (AJCC 7th edition). Epithelial to mesenchymal transition (EMT) phenotype was determined according to expression status of vimentin and E-cadherin, described in a previous study [14].

### Immunohistochemical staining analysis

Sections on microslides were deparaffinized with xylene, hydrated using a diluted alcohol series, and immersed in 0.3% H_2_O_2_ in methanol to quench endogenous peroxidase activity. Sections were treated with TE buffer (10 mM Tris and 1 mM EDTA, pH 9.3) at 98°C for 30 min. To reduce non-specific staining, each section was blocked with 4% bovine serum albumin in PBS with 0.1% Tween 20 for 30 min. The sections were then incubated with anti-Twist1 (1:100, Abcam, Cambridge, UK), anti-SMA (1:100, Millipore, Billerica, MA, USA), anti-FAP (1:100, Abcam, Cambridge, UK), anti-FSP1 (1:100, Millipore, MA, USA), anti-PDGFRα (1:100, Cell Signaling Technology, MA, USA), anti-PDGFRβ (1:100, ABcam, Cambridge, UK) and anti-Tenascin-C (1:100, Abcam, Cambridge, UK) in PBST containing 3 mg/ml goat globulin (Sigma, St. Louis, MO, USA) for 60 min at room temperature, followed by three successive washes with buffer. Sections were then incubated with an anti-mouse/rabbit antibody (Envision plus, Dako, Denmark) for 30 min at room temperature. The chromogen used was 3,3′-diaminobenzidine (Dako, Denmark). Sections were counterstained with Meyer’s hematoxylin. Omitting the primary antibody provided negative controls for immunostaining.

Two pathologists (YHX and SHK) evaluated immunohistochemical results with no prior knowledge of clinicopathological results, and discussed any discrepancies in scores until a consensus was reached. As described in detail previously, immunohistochemical scores for each protein expression were measured semi-quantitatively according to the staining intensity and area and scored 1 to 3. Cases with scores of 2 or 3 were regarded as positive for expression of each protein [4, 5].

### Immunofluorescence imaging intensity assay

Sections on microslides were deparaffinized with xylene, hydrated using a diluted alcohol series, and immersed in 0.3% H_2_O_2_ in methanol to quench endogenous peroxidase activity. Sections were treated with TE buffer (10 mM Tris and 1 mM EDTA, pH 9.3) at 98°C for 30 min. To reduce non-specific staining, each section was blocked with 2% FBS and 1% BSA in PBS for 30 min. The sections were then incubated with primary antibodies overnight at 4°C. The next day, cells were incubated with Alexa Fluor 568 goat anti-mouse IgG (Invitrogen, A12380) and Alexa Fluor 488 goat anti-rabbit IgG (Invitrogen, A11008) secondary antibodies (1:500 dilution) for 1 hr. Nuclei were stained with DAPI and sections were mounted with Vectorshield mounting medium with DAPI for fluorescence detection (Vector lab, H-1200). Fluorescence detection was performed with the Axiovert200II (Carl-Zeiss). The intensity of immunofluorescence of cells was measured using Meta-Morph software.

### Obtaining publicly available gene expression data and gene set enrichment assay

For The Cancer Genome Atlas (TCGA) data, the following “Level 3” processed and normalized gene expression data of 196 esophageal cancer patients (illuminaHiseq RNAseq V2 gene expression, RSEM normalized) were downloaded from the TCGA website. We categorized 40 patients with high levels of Twist1 expression and 39 with low levels of Twist1 expression from 196 esophageal cancer patients included in the TCGA dataset. To identify gene-signature-based differences between Twist1-high/low ESCC populations, we performed GSEA using the Broad Institute’s GSEA tool (http://www.broadinstitute.org/gsea/index.jsp). The gene sets used for GSEA were as follows:

HALLMARK_EPITHELIAL_MESENCHYMAL_TRANSITION(M5930), MISHRA_CARCINOMA_ASSOCIATED_FIBROBLAST_UP (M18292), HALLMARK_ANGIOGENESIS (M5944), HALLMARK_HYPOXIA (M5891), \FINAK_BREAST_CANCER_SDPP_SIGNATURE (M12461), \MESODERM_DEVELOPMENT(M15421).

These gene sets were obtained from the molecular signature database of the Broad Institute (http://software.broadinstitute.org/gsea/msigdb/index.jsp).

### Cell culture and lentiviral transduction

Human esophageal squamous carcinoma TE11 cell line were purchased from RIKEN (Saitama, JAPAN) and were maintained in RPMI medium with high glucose (Life Technologies, Grand Island, NY, USA) supplemented with 10% heat-inactivated fetal bovine serum (Life Technologies), 100 mg/mL penicillin G, and 50 μg/mL streptomycin (Life Technologies) at 37°C in a humidified atmosphere containing 5% CO_2._ 293T cells were purchased from the Korean Cell Line Bank (Seoul, Korea) and cultured in DMEM with 10% fetal bovine serum (FBS).

The packaging of vector was obtained by transfection of 293T. NF #8 cells were transduced with lentivirus-expressing human FLAG-Twist1 and FLAG control vector and selected with hygromycin (Sigma-Aldrich). CAF#8 cells were infected with a lentivirus encoding a non-specific (NS) shRNA or Twist1 shRNA using Polybrene (Millipore) and selected with 2 ug/ml puromycin as decribed previously [14].

### Isolation and culture of fibroblasts

Human esophageal squamous cell carcinoma specimens were obtained from patients undergoing surgery at Samsung Medical Center of SungKyunKwan University of Medicine (Seoul, Korea). An experienced pathologist grossly examined and obtained representative samples of the tumor tissues (CAF, human ESCC CAF) and distal normal tissues (NF, human esophageal normal fibroblast). In detail, fresh tissues obtained from two different areas were cut into small pieces and minced with scalpels in a culture dish. Samples were enzymatically dissociated in 20 ml of D/F12+serum media containing collagenase I in a 37°C incubator for 12−15 h using an orbital shaker. After digestion, samples were centrifuged at 700 rpm for 5 min to separate epithelial cells and fibroblast cells. Fibroblast cells were collected from the supernatant by centrifugation at 800 rpm for 8 min, washed twice with PBS, and cultured in D/F12 media supplemented with 10% FBS and 1% antibiotics.

### Incubation of fibroblasts with conditioned media from ESCC cells

NF (#8, #12) and CAF (#8, #12) were seeded in 10 cm dishes, and after overnight attachment and growth, cells were washed twice with PBS and grown in serum-free D/F12 media. Conditioned medium (CM) was collected after 24 hours (cells reached 70%–80% confluence) and centrifuged at 1,300 rpm for 5 minutes to remove pellet debris. TE11 cells were incubated with CM for 2 days (48 hours).Conditioned media were freshly changed each day.

### Migration and invasion assay

*In vitro* Matrigel invasion assays were done using 6.5-mm Costar transwell chambers (8-μm pore size; Corning, NY, USA). The Transwell filters were coated with appropriate Matrigel (1 mg/ml) (Becton Dickinson, Franklin Lakes, NJ, USA). After the Matrigel solidified at 37°C, 1 × 10^5^ cells were seeded onto the Matrigel. After incubation for 22 hours, the filter was gently removed from the chamber and the noninvasive cells on the upper surface were removed by wiping with a cotton swab. The cells that invaded the Matrigel and attached to the lower surface of the filter were fixed with methanol and stained with H&E solution. The number of cells attached to the lower surface of the polycarbonate filter was counted at ×400 magnification under a light microscope. The migration assay was conducted in the same way as the invasion assay, except for coating with Matrigel. Each type of cell was assayed in triplicate.

### Soft agar colony formation assays

A bottom layer of 0.5 ml of 0.8% agar in DMEM medium containing 10% FBS was prepared and allowed to solidify and cells were seeded in a top layer of 0.4% agar in DMEM medium containing 10% FBS at a density of 5 × 10^2^ cells per well in 24-well plate (duplicates were performed). Cells were incubated at 37°C (medium was added periodically) and colonies formed after 2 weeks were stained with crystal violet and then counted.

### Sphere formation assay

1 × 10^4^ cells were cultured in defined serum-free medium composed of DMEM + F12 medium, 20 ng/ml of EGF (epidermal growth factor; R&D Systems), 20 ng/ml of bFGF (basic fibroblast growth factor; R&D Systems), and B27 supplement (R&D Systems). The cells were seeded in an Ultra-Low Attachment 96 well plate (Corning 3471). Spheroids were resuspended to form secondary and tertiary spheroids. The number of spheroids was counted after 14 days.

### Collagen gel contraction assay

Collagen gel working solution was prepared according to the manufacturer’s instructions (Cell biolabs. Cat.no. CBA-201). Briefly, NFs and CAFs were suspended in the collagen solution (5 × 10^5^cells). The collagen/cell mixture was dispensed into 24well cell culture plate, and the cell-gel mixture was allowed to polymerize at 37°C for 1 hr. After polymerization, 1 ml of culture medium was added to each plate. Collagen embedded cell cultures are incubated for two days, during which stress develops.

### Xenograft experiments

TE11 cells (5 × 10^5^) injected subcutaneously into the right flank of 6-week-old female NOD-SCID mice (Orient Bio, Korea). For coinjection with fibroblasts, TE11 cells and 1.5 × 10^6^ fibroblasts (ENF8 FLAG and ENF8 Twist1) combined, resuspended in100 μl Matrigel/PBS (50:50 mixture) and injected in mice. Animals (six per group) were monitored daily, tumors were measured every week using a digital caliper and the volumes were calculated according the formula: volume = length × width 2/2. Tumors and other organs were fixed overnight at 4°C in formalin (5% in PBS) for histologic analyses. All animal experiments were conducted with the approval of the Institutional Animal Care and Use Committee of Laboratory Animal Research Center at Samsung Biomedical Research Institute.

### Ethics statement

This research complied with the Helsinki Declaration and was approved by the Human Ethics Committee and Research Ethics Committee of Samsung Medical Center. All patients provided written informed consent according to institutional guidelines. Patients were informed that the resected specimens were stored by the hospital and potentially used for scientific research, and that their privacy would be maintained. Follow-up survival data were collected retrospectively through medical record review.

### Statistical analysis

Statistical analyses were conducted using Pearson’s χ^2^ tests, Fisher’s exact tests, Cochan armitage trend test, ANOVA, Mann-Whitney tests, Tukey’s HSD, and Duncan’s test (as a *post hoc* test). Overall survival (OS) and disease free survival (DFS) were determined using the Kaplan-Meier method and the comparison was performed by using the log-rank or Breslow test as appropriate. Survival was measured from the date of surgery. The Cox proportional hazards model was used for multivariate analysis to evaluate the prognostic value of clinicopathologic factors. The hazard ratio (HR) and its 95% confidence interval (CI) were assessed for each factor. All tests were two sided, and *P <* 0.05 was considered statistically significant. All statistical analyses were performed using SPSS software (SPSS Inc., Chicago, IL, USA).

## SUPPLEMENTARY MATERIALS FIGURE



## References

[1] Vannucci L (2015). Stroma as an Active Player in the Development of the Tumor Microenvironment. Cancer Microenviron.

[2] Cirri P, Chiarugi P (2012). Cancer-associated-fibroblasts and tumour cells: a diabolic liaison driving cancer progression. Cancer Metastasis Rev.

[3] Goubran HA, Kotb RR, Stakiw J, Emara ME, Burnouf T (2014). Regulation of tumor growth and metastasis: the role of tumor microenvironment. Cancer Growth Metastasis.

[4] Ha SY, Yeo SY, Xuan YH, Kim SH (2014). The prognostic significance of cancer-associated fibroblasts in esophageal squamous cell carcinoma. PLoS One.

[5] Yang ZT, Yeo SY, Yin YX, Lin ZH, Lee HM, Xuan YH, Cui Y, Kim SH (2016). Tenascin-C, a Prognostic Determinant of Esophageal Squamous Cell Carcinoma. PLoS One.

[6] Lee KW, Kim JH, Han S, Sung CO, Do IG, Ko YH, Um SH, Kim SH (2012). Twist1 is an independent prognostic factor of esophageal squamous cell carcinoma and associated with its epithelial-mesenchymal transition. Ann Surg Oncol.

[7] Yang J, Mani SA, Donaher JL, Ramaswamy S, Itzykson RA, Come C, Savagner P, Gitelman I, Richardson A, Weinberg RA (2004). Twist, a master regulator of morphogenesis, plays an essential role in tumor metastasis. Cell.

[8] Polyak K, Weinberg RA (2009). Transitions between epithelial and mesenchymal states: acquisition of malignant and stem cell traits. Nat Rev Cancer.

[9] Sung CO, Lee KW, Han S, Kim SH (2011). Twist1 is up-regulated in gastric cancer-associated fibroblasts with poor clinical outcomes. Am J Pathol.

[10] Lee KW, Yeo SY, Sung CO, Kim SH (2015). Twist1 is a key regulator of cancer-associated fibroblasts. Cancer Res.

[11] Spaeth EL, Labaff AM, Toole BP, Klopp A, Andreeff M, Marini FC (2013). Mesenchymal CD44 expression contributes to the acquisition of an activated fibroblast phenotype via TWIST activation in the tumor microenvironment. Cancer Res.

[12] Garcia-Palmero I, Torres S, Bartolome RA, Pelaez-Garcia A, Larriba MJ, Lopez-Lucendo M, Pena C, Escudero-Paniagua B, Munoz A, Casal JI (2016). Twist1-induced activation of human fibroblasts promotes matrix stiffness by upregulating palladin and collagen alpha1(VI). Oncogene.

[13] Qin Q, Xu Y, He T, Qin C, Xu J (2012). Normal and disease-related biological functions of Twist1 and underlying molecular mechanisms. Cell Res.

[14] Sung CO, Park CK, Kim SH (2011). Classification of epithelial-mesenchymal transition phenotypes in esophageal squamous cell carcinoma is strongly associated with patient prognosis. Mod Pathol.

