# Supplementary material for: Twist1 is highly expressed in cancer-associated fibroblasts of esophageal squamous cell carcinoma with a prognostic significance
Source: Oncotarget. 2017 May 17;8(39):65265–80. doi: 10.18632/oncotarget.17941 (PMC5630329; doi:10.18632/oncotarget.17941)
Supplement: Supplementary file 1 [file oncotarget-08-65265-s001.pdf]

Twist1 is highly expressed in cancer-associated fibroblasts of esophageal squamous cell carcinoma with a prognostic significance

SUPPLEMENTARY MATERIALS

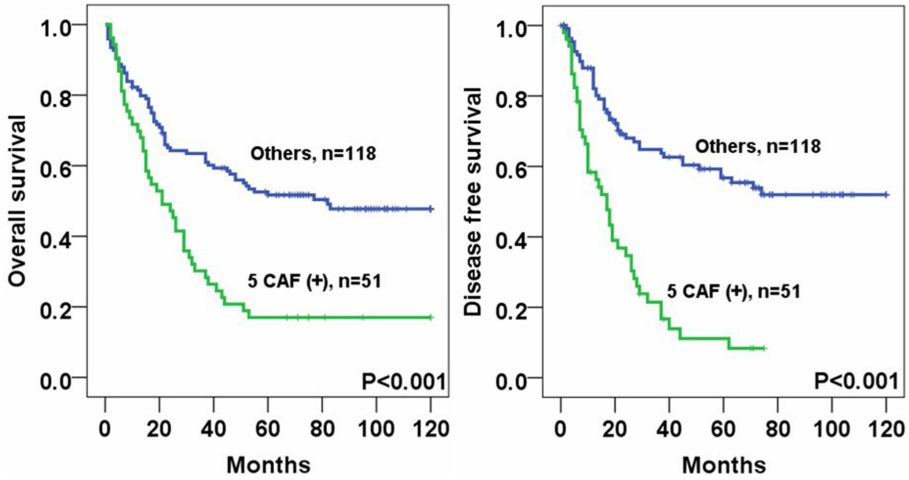

Supplementary Figure 1: Kaplan Meier survival curves of patients with esophageal squamous cell carcinoma (ESCC) according to expression of 5 cancer associated fibroblast (CAF) markers (Twsit1, FSP1, SMA, Tenascin C, PDGFRa). Patients with ESCC showing expression of all 5 CAF markers showed significantly reduced overall survival and disease free survival rate than other patients
